# Peptide–AuNP–drug conjugate-integrated photocrosslinkable hydrogel with pH/glutathione dual-responsive drug delivery and NIR-triggered heating for combined chemo-photothermal treatment

**DOI:** 10.1039/d6ra08612h

**Published:** 2026-10-12

**Authors:** Ranran Wang, Xinyue Wang, Junyu Yi, Yunyan Shi, Gang Wei, Qie Guo

**Affiliations:** a Department of Pharmacy, The Affiliated Hospital of Qingdao University 266003 Qingdao China guoqie822a@qdu.edu.cn; b College of Chemistry and Chemical Engineering, Qingdao University 266071 Qingdao China; c School of Polymer Science and Engineering, Qingdao University of Science and Technology 266042 Qingdao China gweivincent@hotmail.com; d Institute of Biomedical Engineering, College of Life Science, Qingdao University 266071 Qingdao China

## Abstract

Doxorubicin (DOX)-based chemotherapy for breast cancer is frequently compromised by systemic toxicity, poor tumor selectivity, and multidrug resistance, underscoring the need for localized and multimodal therapeutic strategies. Here, we developed an injectable peptide–gold–drug (PAD) conjugate-integrated photocrosslinked methacry-loyl hyaluronic acid (HAMA/PAD) hydrogel that couples stimuli-responsive chemotherapy with NIR-triggered photothermal therapy in a single platform. Two-dimensional peptide nanosheets (PNSs) self-assembled from Fmoc-FKKGSHC served as multifunctional molecular scaffolds for the spatial organization of gold nanoparticles (AuNPs) and thiolated DOX (DOX-SH) through Au–S coordination, generating PAD conjugates. This hierarchical nano-to-macro integration enables the HAMA/PAD hydrogel to function simultaneously as an injectable depot and a localized tumor microenvironment. The resulting HAMA/PAD hydrogel exhibited an interconnected porous architecture, shear-thinning behaviour, compatible with syringe-based delivery prior to photocrosslinking, and a storage modulus of approximately 100 Pa. Importantly, PAD exhibited efficient DOX incorporation, while the hydrogel provided pH- and glutathione (GSH)-responsive drug release, offering stimuli-dependent control over DOX availability. Under 808 nm NIR irradiation, HAMA/PAD rapidly generated localized hyperthermia, reaching approximately 55 °C, and maintained stable photothermal performance over repeated irradiation cycles. Additionally, the hydrogel exhibited good cytocompatibility toward L929 fibroblasts, whereas combined HAMA/PAD and NIR treatment reduced 4T1 breast cancer cell viability to approximately 10%, demonstrating a pronounced chemo-photothermal effect. Overall, this study establishes a PNS-enabled strategy for integrating nanoscale drug/photothermal components within an injectable hydrogel depot, providing a versatile platform for localized, stimulus-responsive, and combined chemo-photothermal treatment.

## Abstract 2

Hydrogels integrated with metallic peptide–drug conjugates demonstrate stimuli responsive drug delivery and NIR-triggered heating for cancer therapy.

## Graphical abstract

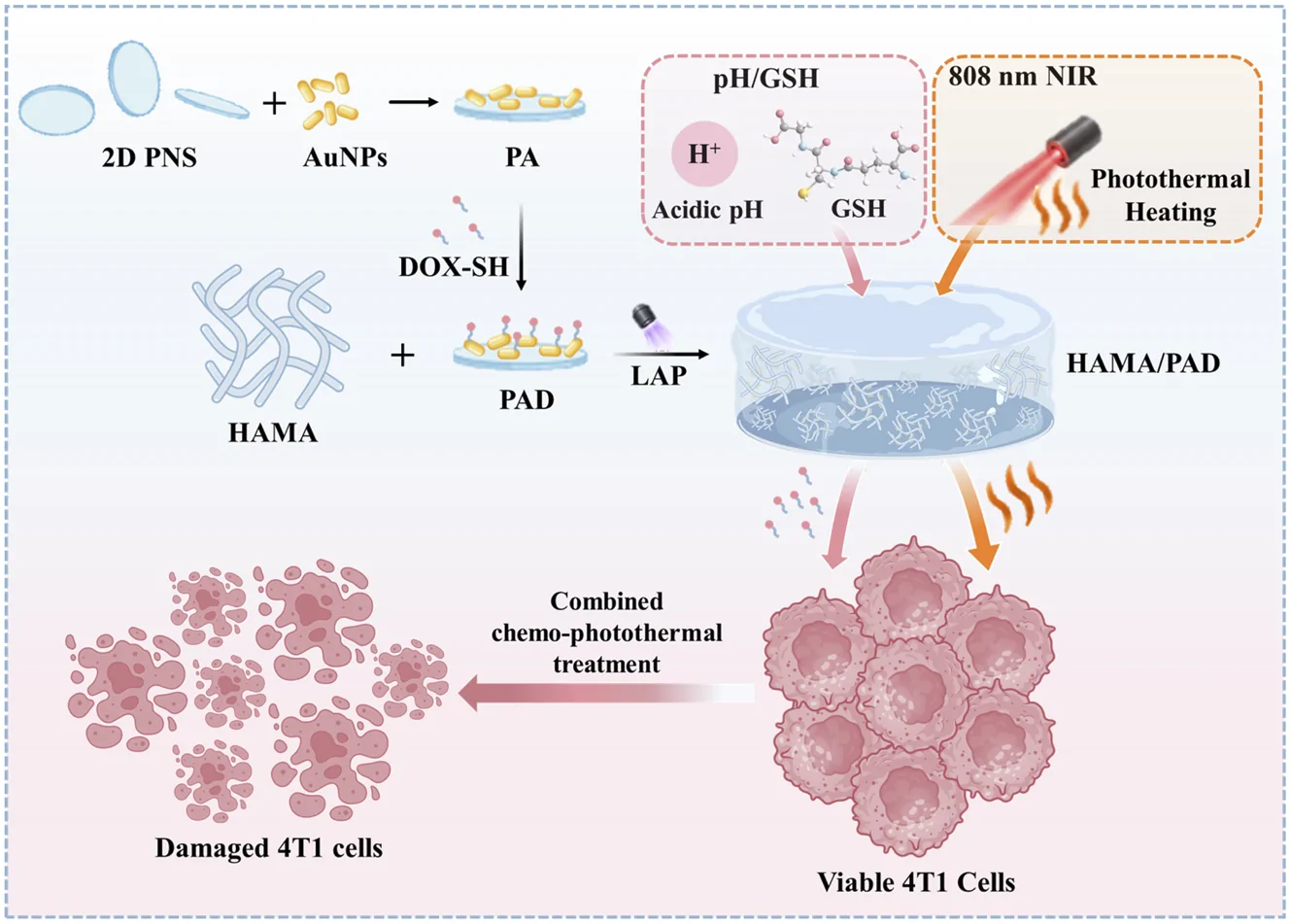

## 1 Introduction

Despite advances in cancer prevention and treatment, breast cancer continues to pose a significant global health challenge because of its high incidence and mortality among women.^1,2^ Although surgical resection and systemic chemotherapy (CT) constitute the cornerstone of breast cancer management, anthracycline-based regimens, especially doxorubicin (DOX), are often limited by severe systemic adverse effects, poor tumor-specific accumulation, and the development of multidrug resistance. These limitations are particularly pronounced in aggressive breast cancer phenotypes, including triple-negative breast cancer.^3–5^ These challenges highlight the need for therapeutic platforms that can simultaneously improve local drug retention, achieve stimulus-controlled drug release, and enhance antitumor efficacy through complementary treatment modalities.

Photothermal therapy (PTT) has attracted considerable attention as a minimally invasive therapeutic strategy, in which photothermal conversion materials (PTCMs) transform near-infrared (NIR) light energy into localized thermal energy for tumor treatment.^6–8^ Its spatiotemporally controllable activation, rapid therapeutic response, and localized cytotoxicity make PTT an attractive complement to conventional CT. Among various PTCMs, gold nanoparticles (AuNPs) are particularly promising because of their favourable biocompatibility, strong localized surface plasmon resonance (LSPR), tunable optical properties, and stable photothermal conversion performance.^8,9^ Nevertheless, PTT as a standalone treatment can be limited by heterogeneous heat distribution, insufficient penetration into deeper tumor regions, and the development of cellular thermotolerance mediated by heat-shock proteins.^10,11^ Combining PTT with CT may overcome these limitations through complementary mechanisms: photothermal heating can directly damage tumor cells while potentially increasing cellular membrane permeability and sensitizing tumor cells to chemotherapeutic agents, whereas CT can eliminate tumor cells that escape thermal ablation.

Tumor tissues exhibit distinct physicochemical characteristics compared with normal tissues, providing endogenous cues for controlled drug loading and release. Notably, solid tumors typically exhibit a mildly acidic extracellular microenvironment, whereas tumor cells maintain markedly elevated intracellular glutathione (GSH) concentrations, which can exceed those of normal cells by several-fold or even an order of magnitude.^12,13^ Such tumor-specific physicochemical features have encouraged the design of stimuli-responsive drug delivery platforms that exploit these endogenous cues to trigger localized drug release, with the aim of enhancing therapeutic efficiency and minimizing systemic or off-target adverse effects.^13,14^ Among these strategies, GSH-responsive systems are particularly attractive for intracellular drug delivery because the high reducing capacity of the cytosol can trigger cleavage or exchange of redox-sensitive linkages, facilitating drug release after cellular uptake.^15,16^ Importantly, exploiting intracellular GSH may also provide an opportunity to modulate the therapeutic behaviour of DOX within tumor cells and improve treatment efficacy against drug-resistant phenotypes.^15,17^ In the present strategy, the use of thiolated DOX (DOX-SH) and Au–S interactions provides a direct molecular basis for constructing a GSH-responsive drug-release mechanism, while the mildly acidic tumor environment may further contribute to stimulus-dependent DOX release.

Peptide-based self-assembled nanostructures have attracted sustained interest for drug delivery applications, owing to their versatile molecular engineering, favorable biocompatibility, and readily tunable functional properties.^18–21^ Compared with one-dimensional (1D) nanofibers or nanotubes, two-dimensional (2D) peptide nanosheets (PNSs) provide broad and accessible interfaces with abundant surface-active sites, favoring the immobilization of metallic nanoparticles and efficient drug loading.^22–25^ In our previous work, the amphiphilic nonapeptide Fmoc-FKKGSHC was designed to self-assemble into uniform 2D PNSs, on which gold nanorods were immobilized for tumor PTT.^26^ Ag_2_S quantum dots were later assembled onto the PNS surface to construct a 2D nanoplatform that combined NIR-II fluorescence imaging and PTT.^27^ We further developed PNS-drug conjugates containing DOX-SH and incorporated them into thermosensitive chitosan hydrogels for GSH-responsive sustained drug delivery.^28^ Furthermore, 1D peptide nanofibers (PNFs) were co-assembled with AuNPs and DOX to form biometallic peptide–drug conjugates. These conjugates were then loaded into oxidized methacrylated hyaluronic acid (O-HAMA) hydrogels for the combined chemo-photothermal therapy of breast cancer.^29^ Bioactive hydrogels derived from hyaluronic acid (HA) have been extensively investigated as local drug delivery platforms, benefiting from their excellent biocompatibility, biodegradability, facile chemical modification, and specific interactions with receptors such as CD44.^30,31^ Injectable hydrogels can further enhance local drug retention and enable minimally invasive administration, making them attractive matrices for localized tumor therapy.^32–35^ In particular, HA-based injectable hydrogel systems incorporating chemotherapeutic agents and photothermal components have recently been explored for localized chemo-photothermal cancer treatment.^31,36^ Incorporating functional peptide-nanoparticle conjugates into such hydrogel matrices therefore offers an opportunity to combine the structural programmability of peptide assemblies, the photothermal properties of inorganic nanomaterials, and the local-delivery capability of hydrogels within a single platform.

Despite growing interest in peptide nanostructures, gold nanomaterials, and injectable hydrogels for cancer therapy, the integration of two-dimensional PNSs with gold–drug conjugates within a photocrosslinkable HA-based hydrogel remains comparatively underexplored. Here, we developed a peptide–Au–drug (PAD) conjugate based on 2D PNSs self-assembled from Fmoc-FKKGSHC and further integrated these conjugates into an injectable photo-crosslinked HAMA hydrogel for localized chemo-photothermal therapy. In contrast to our previously reported 1D PNF-based hydrogel system, the present design employs 2D PNSs as extended interfacial scaffolds for the spatial organization of AuNPs and DOX-SH prior to their incorporation into the photocrosslinked HAMA matrix. As illustrated in Scheme 1, AuNPs were first immobilized on the PNSs to form peptide–Au (PA) nanohybrids, followed by attachment of DOX-SH onto the AuNP surface through Au–S interactions, generating the PAD conjugates. The resulting nanoscale therapeutic assemblies were subsequently incorporated into a photocrosslinkable HAMA network, establishing a hierarchical nano-to-macro therapeutic architecture. In this design, the AuNPs function as NIR-responsive photothermal transducers, while the Au–S bond-mediated immobilization of DOX-SH provides a molecular basis for GSH-responsive drug release through thiol-mediated exchange under reducing conditions. The HAMA matrix further serves as an injectable local depot to retain the therapeutic components at the treatment site and regulate their release. Thus, this platform combines externally triggered PTT with tumor-associated stimulus-responsive CT within a single injectable system. The physicochemical properties, stimulus-responsive drug-release behaviour, photothermal performance, cytocompatibility, and antitumor activity of the HAMA/PAD hydrogel were systematically investigated. This work provides a novel strategy for constructing PNS-enabled injectable biomaterials that integrate molecularly organized drug delivery with multimodal cancer therapy.

**Scheme 1:**
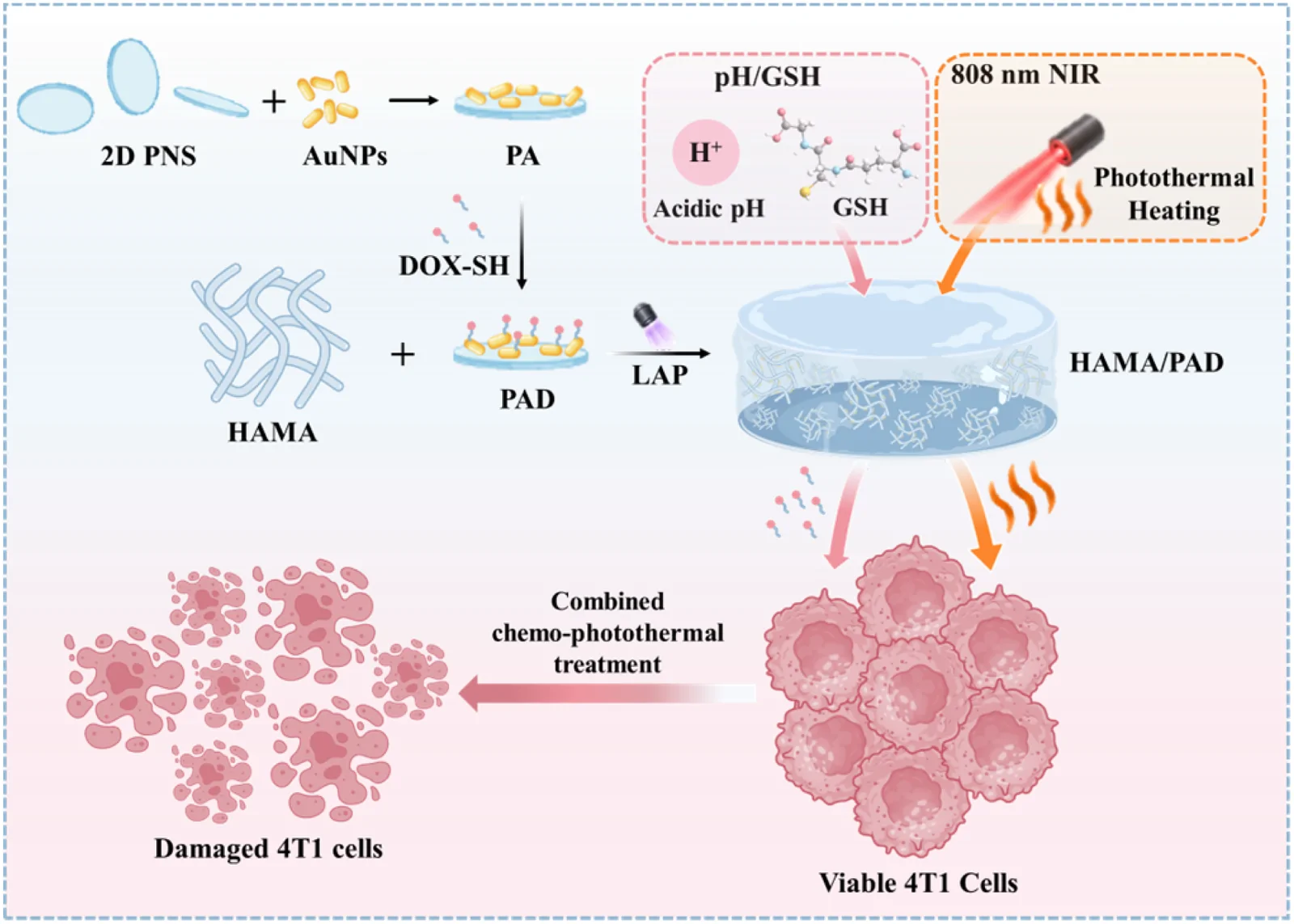
Schematic illustration of the construction of the HAMA/PAD hydrogel and its combined chemo-photothermal treatment.

## 2 Materials and methods

### 2.1 Materials and reagents

Fmoc-FKKGSHC peptide (purity = 95%) was provided by the GenScript Biotech Corporation (Nanjing, China). Ethanol (99.7%), trifluoroacetic acid (TFA, 99%), sodium borohydride (NaBH_4_, 98%), silver nitrate (AgNO_3_, 99.8%), and NaOH (96%) were provided by the Sinopharm Chemical Reagent Co., Ltd. (Beijing, China). 2-Iminothiolane hydrochloride (C_4_H_7_NS HCl, 98%), doxorubicin hydrochloride (DOX, 98%), sodium hyaluronate (HA, *M*_w_ = 400–800 kDa), methacrylic anhydride (MA, 94%), LAP (98%), polyethylene glycol (PEG, *M*_n_ = 10 000), chloroauric acid (HAuCl_4_·3H_2_O, with an Au content of at least 49.0%), hexadecyltrimethylammonium bromide (CTAB, 99%), and ascorbic acid (AA, 99.0%) were provided by Macklin Bio-chemical Co., Ltd. (Shanghai, China). Regenerated cellulose dialysis membranes (MWCO 14 000, 3000, 1000, and 500 Da; width: 34 mm) were acquired from Yibo Bio-technology Co., Ltd. (Hunan, China). Trypsin–EDTA (0.25%) and fetal bovine serum (FBS) were obtained from Gibco. Cell culture was performed using RPMI-1640 and high-glucose DMEM purchased from Solarbio Science & Technology Co., Ltd. (Beijing, China). The calcein-AM/PI live/dead staining and MTT assay kits were supplied by Beyotime Biotechnology Research Institute (Shanghai, China). L929 mouse fibroblasts were purchased from Zhejiang Baidi Biotechnology Co., Ltd. (Jiaxing, Zhejiang, China).

### 2.2 Self-assembly of peptide into PNSs

Peptide self-assembly was initiated by dissolving 5 mg of peptide powder in a 1 : 4 (v/v) mixture of 0.1% TFA aqueous solution and ethanol, yielding a peptide concentration of 1 mg mL^−1^. The resulting solution was maintained at 47 °C for 16 h in a water bath, allowing the peptide molecules to organize into 2D PNSs.

### 2.3 Synthesis of AuNPs

The AuNPs were synthesized by a seed-mediated method. Briefly, the seed solution was prepared by mixing HAuCl_4_·3H_2_O, CTAB, and freshly prepared ice-cold NaBH_4_, followed by incubation at 30 °C for 3 h. For AuNP growth, HAuCl_4_·3H_2_O, AgNO_3_, and CTAB were mixed, followed by the addition of ascorbic acid (AA) and the seed solution. After stirring in the dark for 12 h, the resulting AuNPs were purified by centrifugation (12 000 rpm, 10 min) and repeated washing with deionized water to remove excess CTAB. The purified AuNPs were concentrated through dialysis (MWCO = 3000 Da) against a 30 wt% aqueous PEG solution.

### 2.4 Preparation of PA nanohybrids

The PNS solution was initially subjected to dialysis (MWCO = 500 Da) to decrease its acidity. Subsequently, AuNPs were dispersed in the PNS solution (pH ≈ 7) under rigorous mixing conditions. As a result, the AuNPs were immobilized on the PNS surface, likely facilitated by thiol–gold interactions involving the terminal cysteine residues together with electrostatic and other interfacial interactions, yielding PA nanohybrids.

### 2.5 Preparation of DOX-SH

DOX and 2-iminothiolane hydrochloride were reacted in PBS (pH 7.4) under dark conditions at room temperature for 12 h to generate a sulfur-containing DOX reaction product, hereafter denoted as DOX-SH.

### 2.6 Synthesis of PAD nanohybrids

The PA dispersion was mixed with the DOX-SH reaction solution and stirred for 48 h to promote the association of DOX-SH with the AuNP-containing PA nanohybrids. The DOX-SH reaction solution was quantified on a DOX-equivalent basis. After reaction, the mixture was centrifuged at 10 000 rpm for 10 min to separate the PAD nanohybrids from the unbound drug fraction. The PAD-containing precipitate was collected for subsequent use, whereas the supernatant was retained for quantitative analysis of the unbound DOX-containing fraction.

An aliquot of the recovered supernatant was diluted 20-fold and analysed by UV-vis spectroscopy at 480 nm. The concentration of the unbound DOX-containing fraction was determined using the DOX calibration curve (*C* = 47.66*A* − 2.13) and corrected for the dilution factor. The total amount of unbound drug was calculated from the corrected concentration and the recovered supernatant volume.*m*_free_ = *C*_free_*V*_sup_*m*_loaded_ = *m*_feed_ − *m*_free_
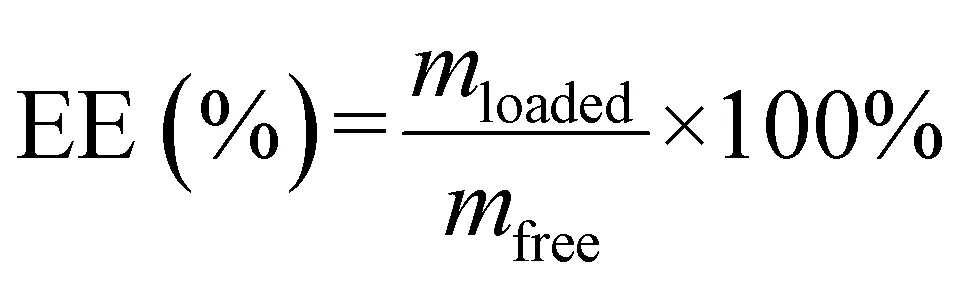

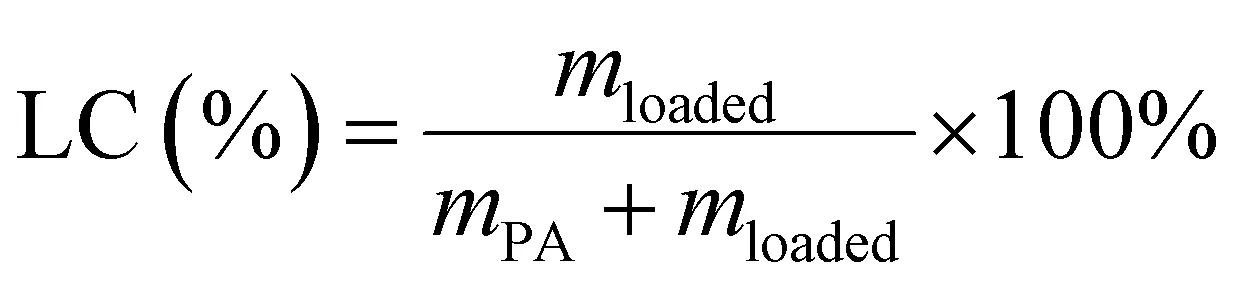
where *m*_feed_ is the initial DOX-equivalent mass used for loading, *m*_free_ is the amount of unbound DOX-equivalent recovered in the centrifugation supernatant, *m*_loaded_ is the calculated amount of loaded drug, and *m*_PA_ is the total mass of the PA carrier. Three independent PAD loading experiments were performed, and the results are expressed as mean ± SD (*n* = 3). PA and PAD were maintained as aqueous dispersions throughout the preparation and were not isolated as completely dried intermediates; therefore, the reported EE and LC values describe drug incorporation rather than the absolute gravimetric synthetic recovery yield of PA or PAD.

### 2.7 Preparation of the HAMA/PAD composite hydrogel

HAMA was prepared by reacting 2 wt% HA aqueous solution (80 mL) with MA (4.8 mL) in the presence of 5.0 M NaOH (2.4 mL). The reaction was conducted at 0 °C for 24 h under continuous stirring. The resulting product was purified by dialysis against deionized water (MWCO = 14 000 Da) for 4 days and subsequently freeze-dried. The obtained HAMA was stored at 4 °C before use.

For hydrogel fabrication, 50 mg of HAMA was dissolved in 1 mL of PAD dispersion, corresponding to a HAMA concentration of 5 wt%. LAP (2.5 mg) was then added, corresponding to 0.25 wt%. The precursor was subsequently irradiated with 365 nm UV light for 30 s to initiate photopolymerization and form the HAMA/PAD composite hydrogel.

### 2.8 Swelling test

The precursor was cast into cylindrical molds and photopolymerized under UV irradiation to obtain three size-matched hydrogel specimens. Their initial masses were recorded as *W*_0_. Each specimen was then placed in an equal volume of PBS with a designated pH of 5.0, 6.2, or 7.4. At selected intervals, the hydrogels were withdrawn, gently blotted with filter paper to remove excess surface liquid, and weighed to obtain *W*_*i*_.

The swelling index was calculated as: swelling index (%) = (*W*_*i*_ − *W*_0_)/*W*_0_ × 100%.

### 2.9 Degradation test

Three hydrogel samples of identical size were prepared by UV irradiation of the pre-gel solution in cylindrical molds. After reaching equilibrium swelling in PBS (pH 5.0, 6.2, or 7.4; equal volumes), the initial mass *W*_0_ was recorded. The buffer was refreshed every other day, and the residual mass *W*_t_ was measured at predetermined intervals.

The residual mass was calculated as: residual mass (%) = *W*_t_/*W*_0_ × 100%.

### 2.10 *In vitro* drug release

Aliquots of the HAMA/PAD hydrogel (300 µL) were placed in the inner chambers of ultrafiltration tubes (MWCO = 3000 Da), and 20 mL of PBS at pH 7.4, 6.2, or 5.0 (containing 0 or 2 mM GSH) was added. At predetermined intervals, a fixed volume of the release medium was collected for UV-vis measurement and immediately replaced with the same volume of fresh PBS supplemented with the corresponding GSH concentration. DOX-SH release was quantified by monitoring its cumulative amount using UV-vis spectrophotometry.

### 2.11 Photothermal test

Aliquots (0.5 mL) of each sample were placed in 1.5 mL tubes and exposed to 808 nm laser irradiation. Temperature changes were monitored at predefined intervals using a thermal imaging camera, and the resulting data were used to generate temperature–time profiles and photothermal heating curves. The photothermal curves of AuNPs at different concentrations and laser powers, as well as those of different samples at 1.5 W cm^−2^, were measured, and infrared thermal images were captured at various time intervals. For the photothermal cycling stability test, 0.5 mL of HAMA/PAD was irradiated at 1.5 W cm^−2^ for three cycles of 5 min heating and 5 min cooling, and the temperature change in each cycle was recorded.

### 2.12 Cell culture

L929 fibroblasts and 4T1 mammary carcinoma cells were maintained in either RPMI-1640 or high-glucose DMEM supplemented with 10% FBS and 1% penicillin–streptomycin. Cell cultures were kept at 37 °C under humidified conditions with 5% CO_2_ and passaged every 2–3 days.

### 2.13 Cytocompatibility

L929 fibroblasts were dispensed into 96-well plates at 1 × 10^4^ cells per well and incubated overnight to allow cell attachment. Subsequently, the cells were incubated with AuNPs at escalating concentrations (0–250 µg mL^−1^) or with the various formulations (HAMA, HAMA/PNS, HAMA/PA, and HAMA/PAD, with or without NIR irradiation) for durations of 24, 48, or 72 h. The MTT assay was employed to evaluate cellular metabolic activity, with the optical density of each sample determined at 570 nm. Cell viability was further visualized by dual staining with calcein-AM and PI following the manufacturer's protocol, and the stained cells were examined using an inverted fluorescence microscope.

### 2.14 Determination of antitumor activity *in vitro*

4T1 cells during the exponential growth phase were plated in 96-well plates at a density of 1 × 10^4^ cells per well and incubated overnight to facilitate cell attachment. The cells were subjected to one of six distinct experimental interventions: (i) untreated control (cultured in standard medium only), (ii) NIR irradiation alone, (iii) HAMA/PNS nanocomposite treatment, (iv) HAMA/PA nanocomposite treatment, (v) HAMA/PAD nanocomposite treatment, or (vi) HAMA/PAD nanocomposite combined with NIR irradiation. For all NIR-exposed groups, photothermal stimulation was delivered *via* a focused 808 nm NIR laser with an intensity of 1.5 W cm^−2^ for exactly 5 min-ensuring uniform, reproducible energy deposition across wells. Following 72 h of incubation, cellular viability was determined using the MTT assay, while calcein-AM/PI dual staining was performed to quantify the proportions of viable and nonviable cells.

### 2.15 Characterization techniques

The physicochemical features of the fabricated materials were examined using complementary microscopic, spectroscopic, thermal, rheological, and elemental analysis techniques. AFM (FM-Nanoview 6800, FSM Precision Instruments Co., Ltd., Suzhou, China) was used to determine the morphology and thickness of the peptide nanosheets, whereas TEM (JEM-1400Flash, JEOL Ltd., Tokyo, Japan) provided morphological information on the PNSs, AuNPs, PA, and PAD. The internal architecture of freeze-dried hydrogel samples was visualized by SEM (VEGA3, TESCAN, Brno, Czech Republic). Optical absorption characteristics were analyzed using a UV-vis spectrophotometer (SPECORD® 210 PLUS, Analytik Jena AG, Jena, Germany), and chemical structures were examined by FTIR spectroscopy (Nicolet iS50, Thermo Fisher, MA, USA). The mechanical and flow properties of the hydrogels were investigated with a rotational rheometer (MCR 302, Anton Paar, Graz, Austria), including strain-dependent viscoelasticity and viscosity as a function of shear rate. Temperature variations during NIR exposure were monitored using an infrared thermal camera (FOTRIC 220S, FOTRIC, China). Calcein-AM/PI fluorescence images were collected with an inverted fluorescence microscope (CKX53, Olympus, Tokyo, Japan). The elemental composition of the samples was determined using an inductively coupled plasma instrument (Avio 200, PerkinElmer, USA). X-ray photoelectron spectroscopy (XPS) measurements were performed using an ESCALAB Xi+ X-ray photoelectron spectrometer (Thermo Fisher Scientific, USA). ^1^H NMR spectra were recorded on a 400 MHz NMR spectrometer (Bruker, Germany) using D_2_O as the solvent.

### 2.16 Statistical analysis

Quantitative data are presented as mean ± SD from three independent experiments unless otherwise specified. Comparisons between two groups were performed using Student's *t*-test. Comparisons among multiple groups were performed using one-way ANOVA followed by Tukey's *post hoc* multiple-comparison test. A value of *p* < 0.05 was considered statistically significant.

## 3 Results and discussion

### 3.1 Synthesis and morphological observation of PA nanohybrids

2D PNSs were prepared through the self-assembly of the amphiphilic nonapeptide Fmoc-FKKGSHC in an ethanol/0.1% TFA aqueous solution (4 : 1, v/v) at 47 °C for 16 h. As illustrated in Fig. 1a, the peptide was rationally designed with distinct functional moieties that contribute to both nanosheet formation and subsequent functionalization. The *N*-terminal fluorenylmethoxycarbonyl (Fmoc) group provides an aromatic surface capable of π–π stacking, while the lysine residues contribute charged side chains that facilitate intermolecular interactions and surface hydration. Importantly, the terminal cysteine residue contains a thiol group with a strong affinity for gold surfaces, providing a molecular anchoring site for subsequent AuNP immobilization and Au–S-mediated drug conjugation. The development of the 2D PNS architecture is likely governed by the synergistic interplay of π–π stacking, hydrogen bonding, hydrophobic association, and electrostatic interactions between the peptide molecules. Thus, the self-assembled PNSs not only provide a defined 2D nanostructure but also serve as a multifunctional molecular scaffold for the hierarchical construction of the PA and PAD conjugates.

**Fig. 1:**
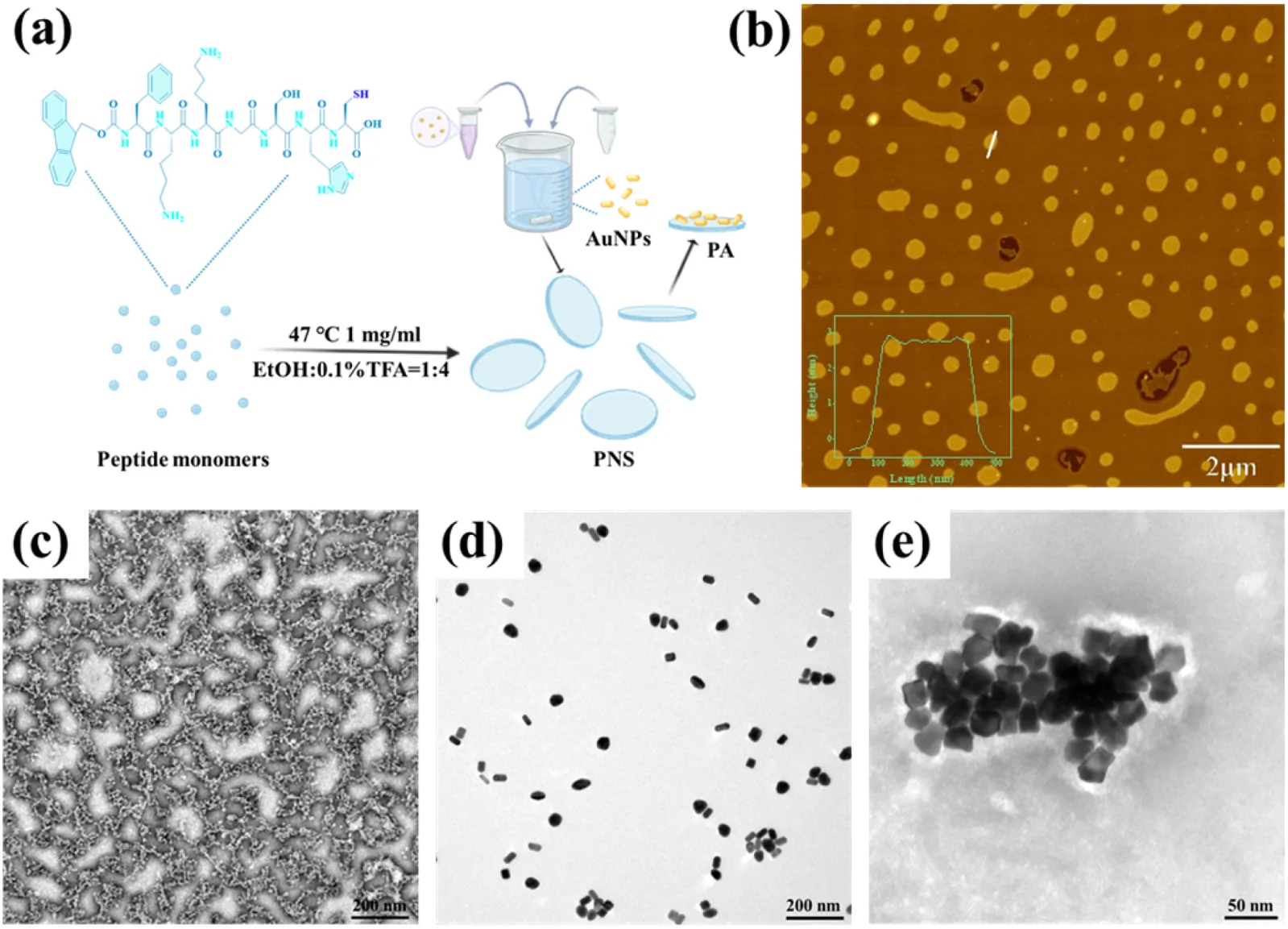
(a) Schematic synthesis of PA through peptide self-assembly and subsequent AuNPs immobilization. (b) AFM image of PNSs with the corresponding height profile (inset). (c) TEM image of PNSs. (d) TEM image of AuNPs. (e) TEM image of PA nanohybrids.

AFM imaging revealed well-defined nanosheet-like structures with lateral dimensions in the micrometer range and an average thickness of approximately 3 nm, as determined from the corresponding height profile (Fig. 1b). TEM imaging further confirmed the thin, lamellar morphology of the PNSs (Fig. 1c), demonstrating the successful formation of 2D peptide assemblies. The synthesized AuNPs exhibited relatively uniform particle sizes of approximately 20–30 nm, with predominantly quasi-spherical to short rod-like morphologies (Fig. 1d). Following incubation with the PNSs, AuNPs were clearly distributed on the nanosheet surfaces (Fig. 1e), supporting the formation of PA nanohybrids.

This surface association is likely promoted by the strong affinity between the thiol groups of the terminal cysteine residues and the gold surface, together with complementary electrostatic and interfacial interactions. Notably, the ultrathin 2D architecture of the PNSs provides a large and readily accessible interfacial area for AuNP immobilization, which may facilitate efficient exposure of the gold surface for subsequent DOX-SH conjugation. Meanwhile, nanoscale organization of AuNPs on the peptide scaffold can help establish a spatially integrated photothermal and drug-delivery interface. These structural features demonstrate that the PNSs function not merely as passive carriers but as molecularly designed scaffolds for hierarchical assembly of the PAD nanoplatform.^18^

### 3.2 Preparation and spectroscopic characterization of PAD nanohybrids

To elucidate the interactions among the individual components and verify the stepwise construction of the PAD nanohybrids, the optical and structural characteristics of the intermediate and final products were systematically analyzed. As illustrated in Fig. 2a, DOX was reacted with 2-iminothiolane to generate a sulfur-containing DOX reaction product (denoted as DOX-SH), which was directly used in the subsequent PAD preparation. The resulting sulfur-containing DOX reaction product was subsequently associated with the AuNP surfaces of PA through Au–S-mediated interactions to form PAD.

**Fig. 2:**
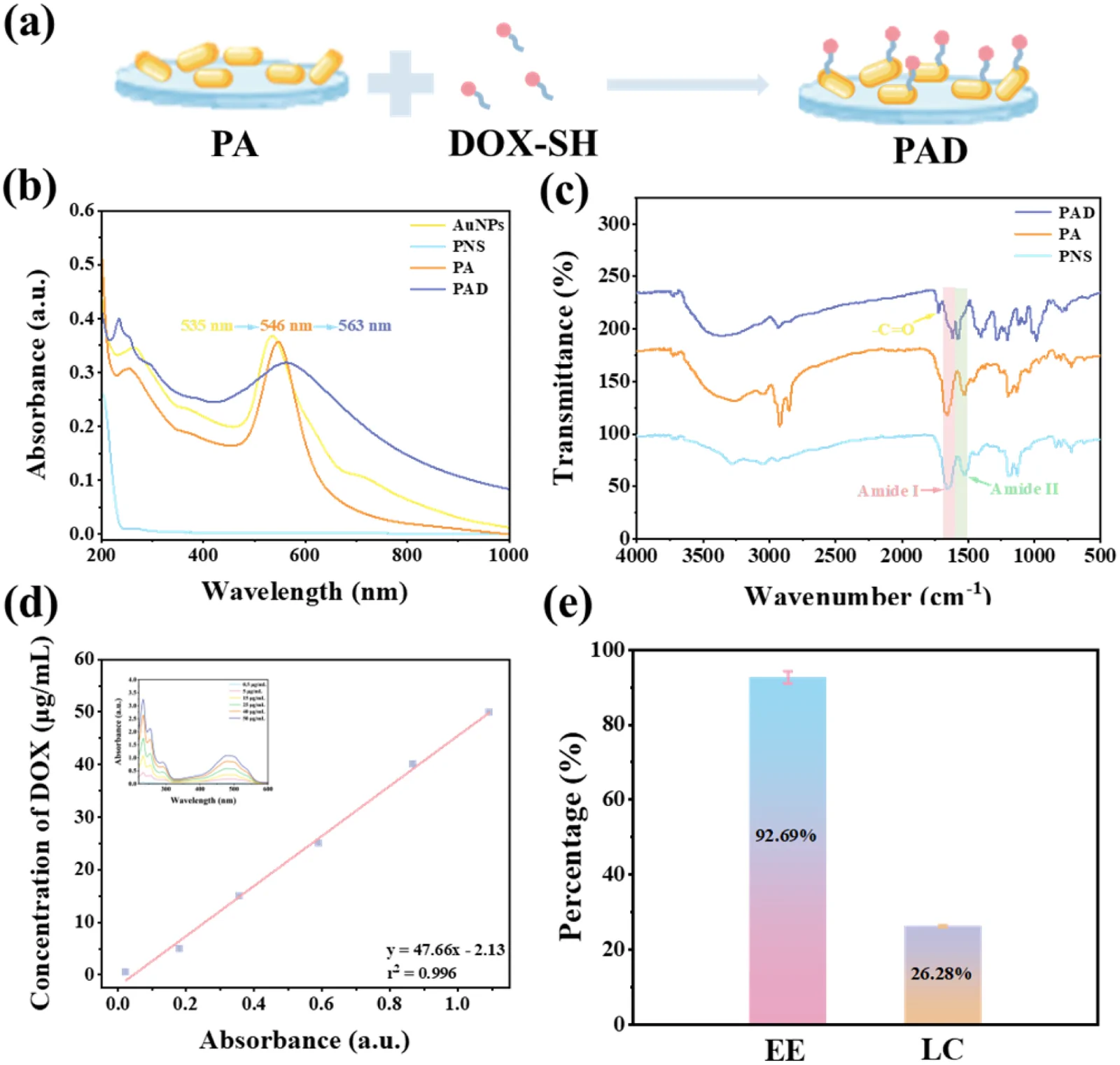
(a) Synthesis procedure of PAD. (b) UV-vis absorption spectra of PNS, PA, AuNPs, and PAD. (c) FTIR spectra of PNS, PA, and PAD. (d) Calibration curve of DOX for quantitative analysis. The inset shows the UV-vis absorption spectra of DOX solutions at different concentrations. (e) Encapsulation efficiency (EE) and drug loading content (LC) of DOX in the PAD nanohybrid determined by quantification of the unbound DOX-containing fraction in the centrifugation supernatant. Data are presented as mean ± SD (*n* = 3 independent loading experiments).

As shown in Fig. 2b, the pristine AuNPs displayed a distinct localized surface plasmon resonance (LSPR) band centered at around 535 nm. Following immobilization onto the PNS surface, the LSPR maximum shifted to approximately 546 nm, consistent with a change in the local dielectric environment and interfacial interactions surrounding the AuNPs.^6,9^ After subsequent conjugation with DOX-SH, the LSPR peak further red-shifted to approximately 563 nm. This additional spectral shift suggests a further alteration of the local surface environment of the AuNPs following DOX-SH attachment. Although LSPR shifts alone do not definitively establish the chemical nature of each interaction, the sequential spectral changes, together with the designed Au–S chemistry, provide supportive evidence for the stepwise formation of PA and PAD.

FTIR spectroscopy was further employed to characterize the sequential assembly of the nanoplatform (Fig. 2c). PNS exhibited characteristic amide I (≈1650 cm^−1^) and amide II (≈1530 cm^−1^) bands, reflecting the presence of the peptide backbone. After hybridization with AuNPs, PA retained these characteristic amide bands, indicating that the major structural features of the peptide nanosheets were maintained during AuNP immobilization. Compared with PA, PAD exhibited enhanced C=O stretching absorption around 1720 cm^−1^, together with noticeable changes in the fingerprint region (1300–1000 cm^−1^), particularly in the C–O/C–O–C-related vibrational bands. These spectral changes are consistent with the introduction of DOX-SH into the PA hybrid. Collectively, the progressive LSPR shifts and FTIR spectral changes support the successful formation of PAD while indicating retention of the major structural features of the peptide component.

To further investigate the chemical state of sulfur during PAD formation, additional XPS analysis was performed (Fig. S1). The high-resolution S 2p spectrum of DOX-SH exhibited a characteristic doublet at 163.45 eV (S 2p_3/2_) and 164.60 eV (S 2p_1/2_). After incorporation into the AuNP-containing PAD nanohybrid, the corresponding peaks shifted to 162.94 and 164.09 eV, respectively, representing a decrease of approximately 0.51 eV in binding energy. The S 2p spin–orbit separation remained approximately 1.15 eV, with an S 2p_3/2_ : S 2p_1/2_ area ratio close to 2 : 1. The shift toward lower binding energy indicates an altered electronic environment of sulfur and is consistent with the presence of Au-associated sulfur species in PAD. These XPS results therefore support Au–S-mediated coordination during PAD formation.

DOX loading was quantitatively evaluated using the UV-vis calibration curve shown in Fig. 2d. The concentration of the unbound DOX-containing fraction in the centrifugation supernatant was determined at 480 nm after correction for the 20-fold dilution factor, and the total amount of unbound drug was calculated by taking the recovered supernatant volume into account. Based on three independent loading experiments, the EE and LC of PAD were determined to be 92.69 ± 1.61% and 26.28 ± 0.34%, respectively (Fig. 2e, Table S1). These results indicate efficient incorporation of the DOX-containing component into the PA nanohybrid under the tested loading conditions. The relatively high EE may be associated with the accessible Au-containing interfaces provided by the PA nanohybrid, which offer multiple potential binding and association sites for DOX-SH.

### 3.3 Preparation and physicochemical properties of the HAMA/PAD hydrogel

The fabrication of the HAMA/PAD hydrogel is schematically illustrated in Fig. 3a. HA was first methacrylated to introduce photocrosslinkable methacryloyl groups, yielding HAMA, which was subsequently mixed with the PAD nanohybrids and HAMA-specific photoinitiator, lithium phenyl-2,4,6-trimethylbenzoylphosphinate (LAP). Upon exposure to 365 nm UV irradiation, the methacryloyl groups underwent free-radical polymerization, generating a three-dimensional (3D) crosslinked HAMA network.^32^ During this process, the PAD nanohybrids were physically entrapped and distributed within the polymer matrix, forming an integrated nano-micro composite hydrogel.

**Fig. 3:**
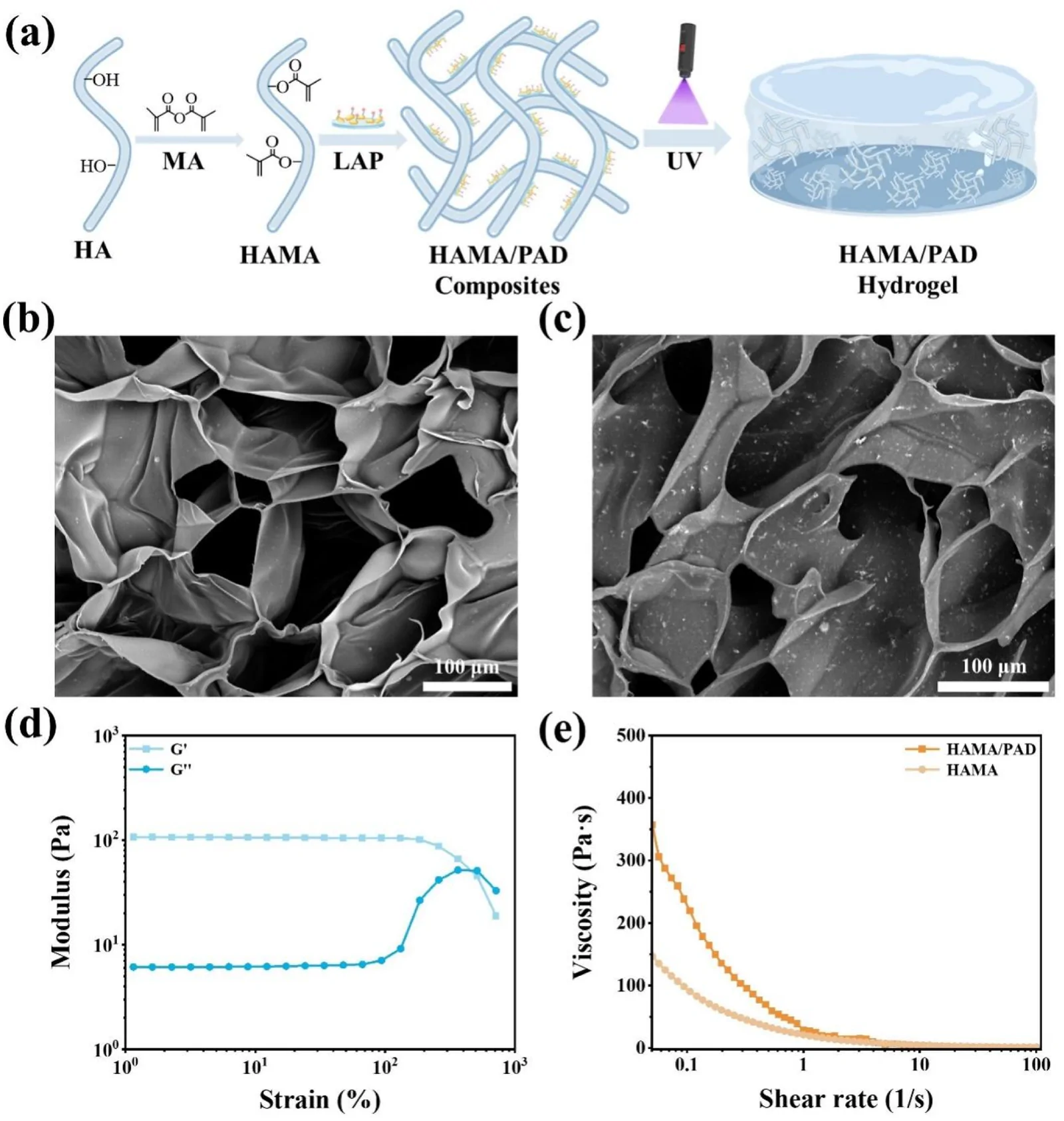
(a) Synthesis procedure of HAMA/PAD hydrogel. (b, c) SEM images of freeze-dried. (b) HAMA and (c) HAMA/PAD hydrogels. (d) Storage modulus (*G*′) and loss modulus (*G*″) of HAMA/PAD hydrogel. (e) Shear rate-dependent viscosity of HAMA and HAMA/PAD precursor solutions.

The successful methacrylation of HA was further verified by ^1^H NMR spectroscopy (Fig. S2). Compared with native HA, HAMA exhibited two new resonances at approximately 5.61 and 6.06 ppm, corresponding to the vinyl protons of methacryloyl groups. Based on the integrated vinyl proton signals relative to the HA backbone proton region, the degree of methacrylation was calculated to be approximately 34.2%, confirming successful introduction of photocrosslinkable methacryloyl groups onto the HA backbone.

SEM images of the freeze-dried samples revealed interconnected porous architectures in both HAMA and HAMA/PAD hydrogels, with pore dimensions ranging from tens to hundreds of micrometers (Fig. 3b and c). Compared with pristine HAMA, HAMA/PAD exhibited numerous particulate features distributed along the pore walls, consistent with the successful incorporation of PAD into the hydrogel network. The overall interconnected architecture was largely retained after PAD incorporation, suggesting that the nanohybrids did not substantially disrupt formation of the HAMA network. Such an open porous structure is favorable for water penetration and molecular transport, which may facilitate diffusion of small molecules and enable interaction between the incorporated PAD nanohybrids and the surrounding aqueous environment. Importantly, confinement of PAD within the crosslinked HAMA matrix may also reduce the mobility and aggregation of the nanoscale components while providing a hydrated 3D depot for localized drug release.

Rheological characterization further demonstrated the gel-like and shear-responsive properties of HAMA/PAD. As shown in Fig. 3d, within the low-strain linear viscoelastic region, the storage modulus (*G*′) remained approximately 100 Pa and was markedly higher than the loss modulus (*G*″, approximately 6 Pa), indicating predominantly elastic, gel-like behaviour. With increasing strain, *G*′ decreased while *G*″ increased, suggesting progressive disruption and yielding of the hydrogel network. As shown by the shear-rate profiles (Fig. 3e), both HAMA and HAMA/PAD exhibited a pronounced reduction in apparent viscosity as the shear rate increased, indicative of characteristic shear-thinning behaviour. HAMA/PAD exhibited a consistently higher viscosity than HAMA over the tested shear-rate range, which may be associated with additional physical interactions, including chain entanglement and hydrogen bonding between PAD and HAMA. These rheological results demonstrate the gel-like viscoelastic behaviour of the photocrosslinked HAMA/PAD hydrogel and the shear-thinning characteristics of its precursor solution, which are compatible with syringe-based delivery prior to photocrosslinking.

### 3.4 Swelling, degradation, and drug release behaviour of HAMA/PAD hydrogel

Swelling and degradation behaviours are important characteristics of injectable hydrogels because they influence water uptake, structural stability, and the diffusion and release of therapeutic agents. As shown in Fig. 4a, The HAMA/PAD hydrogel underwent pronounced swelling within the initial 24 h, after which the swelling ratio increased progressively at a slower rate during the following days. The swelling degree was moderately enhanced under acidic conditions, with the pH 5.0 group exhibiting the highest water uptake. This pH-dependent behaviour may be related to changes in the ionization state and hydration of functional groups within the HAMA/PAD network, together with increased relaxation of polymer chains under acidic conditions.

**Fig. 4:**
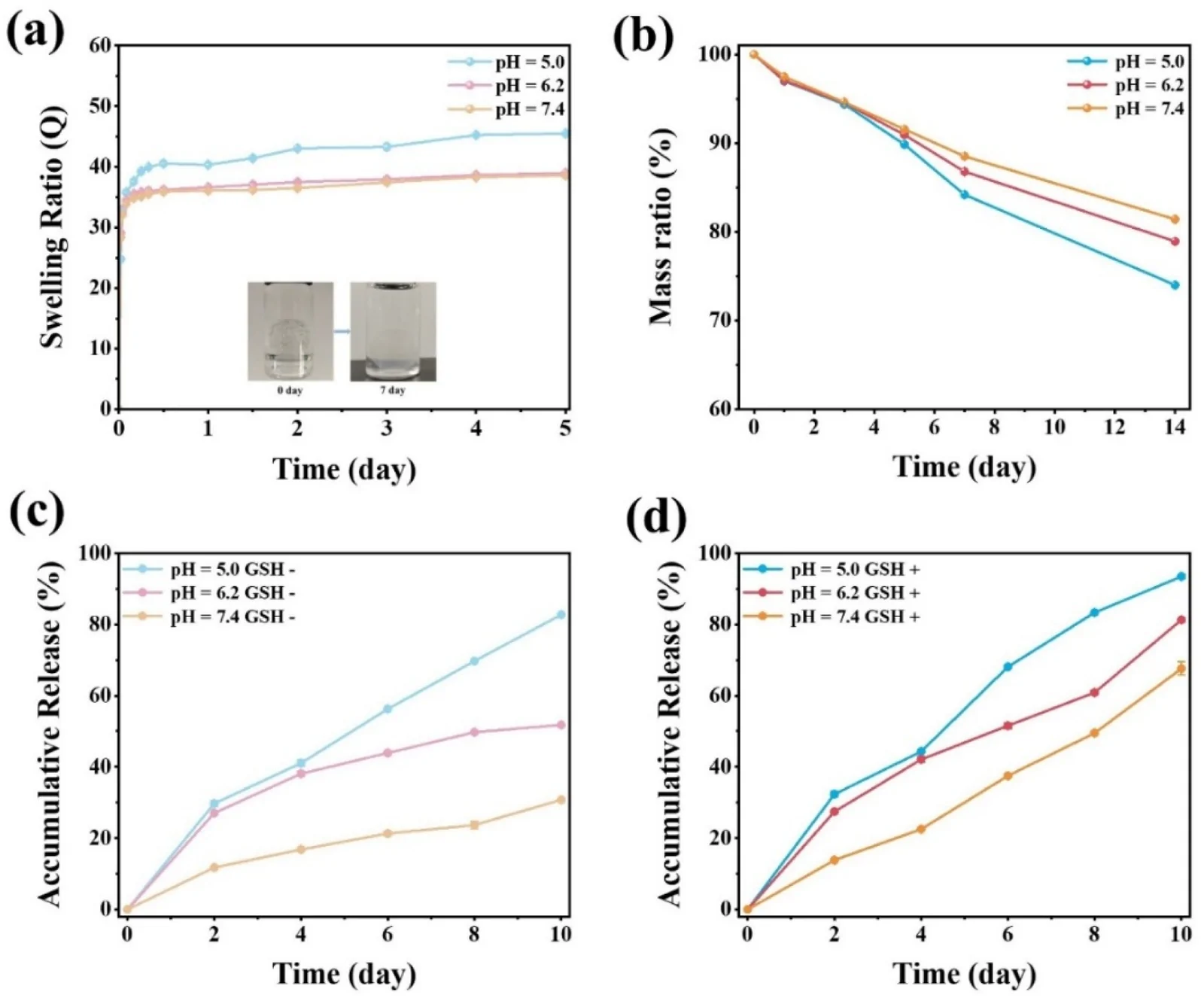
(a) Swelling behavior under different pH conditions. Representative photographs of the hydrogel before and after swelling are shown in the inset. (b) *In vitro* degradation behavior under different pH conditions. (c) DOX release kinetics at varying pH conditions in the absence of GSH. (d) pH-responsive DOX release profiles obtained in a medium supplemented with 2 mM GSH.

The degradation profiles further demonstrated a gradual loss of hydrogel mass over 14 days, with residual mass fractions of approximately 74%, 79%, and 81% at pH 5.0, 6.2, and 7.4, respectively (Fig. 4b). The greater mass loss at lower pH suggests that the HAMA-based network undergoes moderately accelerated degradation under acidic conditions, potentially due to acid-facilitated cleavage or hydrolysis of susceptible chemical linkages. Importantly, the retained mass of more than 70% after 14 days under all tested conditions indicates that the hydrogel maintains substantial structural integrity over the investigated period. The combination of gradual degradation and pH-dependent swelling may be advantageous for localized drug delivery, allowing the hydrogel to maintain a relatively stable depot while permitting progressive water penetration and molecular transport. Moreover, the enhanced swelling and degradation observed under acidic conditions may be relevant to the mildly acidic tumor microenvironment, although the specific contribution of pH to *in vivo* drug release and degradation would require further investigation.

The DOX-release profiles of the HAMA/PAD hydrogel under different pH and redox conditions are shown in Fig. 4c and d. In the absence of GSH, cumulative DOX release over 10 days reached approximately 83%, 52%, and 31% at pH 5.0, 6.2, and 7.4, respectively (Fig. 4c), demonstrating a pronounced pH-dependent release behavior. The pronounced increase in drug release under acidic conditions can be attributed to the synergistic effects of accelerated hydrogel swelling and matrix degradation, increased hydration and relaxation of the polymer network, and consequently facilitated diffusion of DOX-containing components.

The approximately 31% cumulative release observed at pH 7.4 in the absence of GSH indicates that the loaded DOX-containing species are unlikely to be retained exclusively through strong Au–S interactions. A fraction of DOX-SH may remain physically entrapped within the HAMA matrix or weakly associated with the peptide/AuNP interface and can therefore undergo gradual diffusion or desorption during prolonged incubation. Thus, the GSH-induced enhancement in release is considered an additional thiol-mediated release mechanism superimposed on this baseline diffusion/desorption process. Notably, the presence of 2 mM GSH further increased cumulative DOX release to approximately 93%, 81%, and 68% at pH 5.0, 6.2, and 7.4, respectively (Fig. 4d). The additional GSH-dependent release is consistent with the designed Au–S-mediated drug immobilization mechanism. GSH contains a highly nucleophilic thiol group that can interact strongly with the AuNP surface and compete with Au-bound DOX-SH, thereby promoting thiol exchange and dissociation of DOX-SH from the gold surface. This redox-responsive mechanism provides an additional level of control beyond the pH-dependent behaviour of the hydrogel matrix. Importantly, the combination of enhanced release under acidic conditions and further GSH-triggered release is relevant to the physicochemical properties of the tumor microenvironment.

Collectively, these data demonstrate that HAMA/PAD possesses dual pH- and GSH-responsive DOX-release characteristics. The HAMA matrix provides a relatively stable local drug reservoir, while the Au–S-mediated PAD architecture introduces an additional molecular mechanism for stimulus-responsive drug liberation. Such hierarchical control over drug release may help improve local DOX availability while limiting premature drug loss under more physiological conditions, supporting the potential of HAMA/PAD as a localized chemo-photothermal therapeutic platform.

### 3.5 NIR-responsive photothermal performance

AuNPs are widely investigated as photothermal conversion agents because their LSPR enables efficient conversion of absorbed optical energy into heat.^6,7,9^ As indicated by the UV-vis spectra in Fig. 2b, the Au-containing formulations exhibited characteristic LSPR absorption in the visible region together with a broad absorption tail extending into the NIR region. This optical response provides a basis for activating the system using 808 nm NIR irradiation.

The photothermal performance of AuNPs was first investigated as a function of nanoparticle concentration. As shown in Fig. 5a, upon exposure to 808 nm NIR light at 1.5 W cm^−2^, AuNP dispersions containing 100, 150, and 200 µg mL^−1^ AuNPs showed pronounced, concentration-dependent photothermal heating. After 300 s of irradiation, the temperatures reached approximately 49, 53, and 58 °C, respectively. The progressive increase in temperature with AuNP concentration can be attributed to the greater amount of NIR-absorbing photothermal material available for light-to-heat conversion. The effect of irradiation intensity was subsequently evaluated by fixing the AuNP concentration at 150 µg mL^−1^. As shown in Fig. 5b, a progressive elevation in laser power density from 1.25 to 2.00 W cm^−2^ substantially enhanced photothermal heating, with the terminal temperature rising from approximately 40 to 80 °C, demonstrating a clear power-dependent photothermal response.

**Fig. 5:**
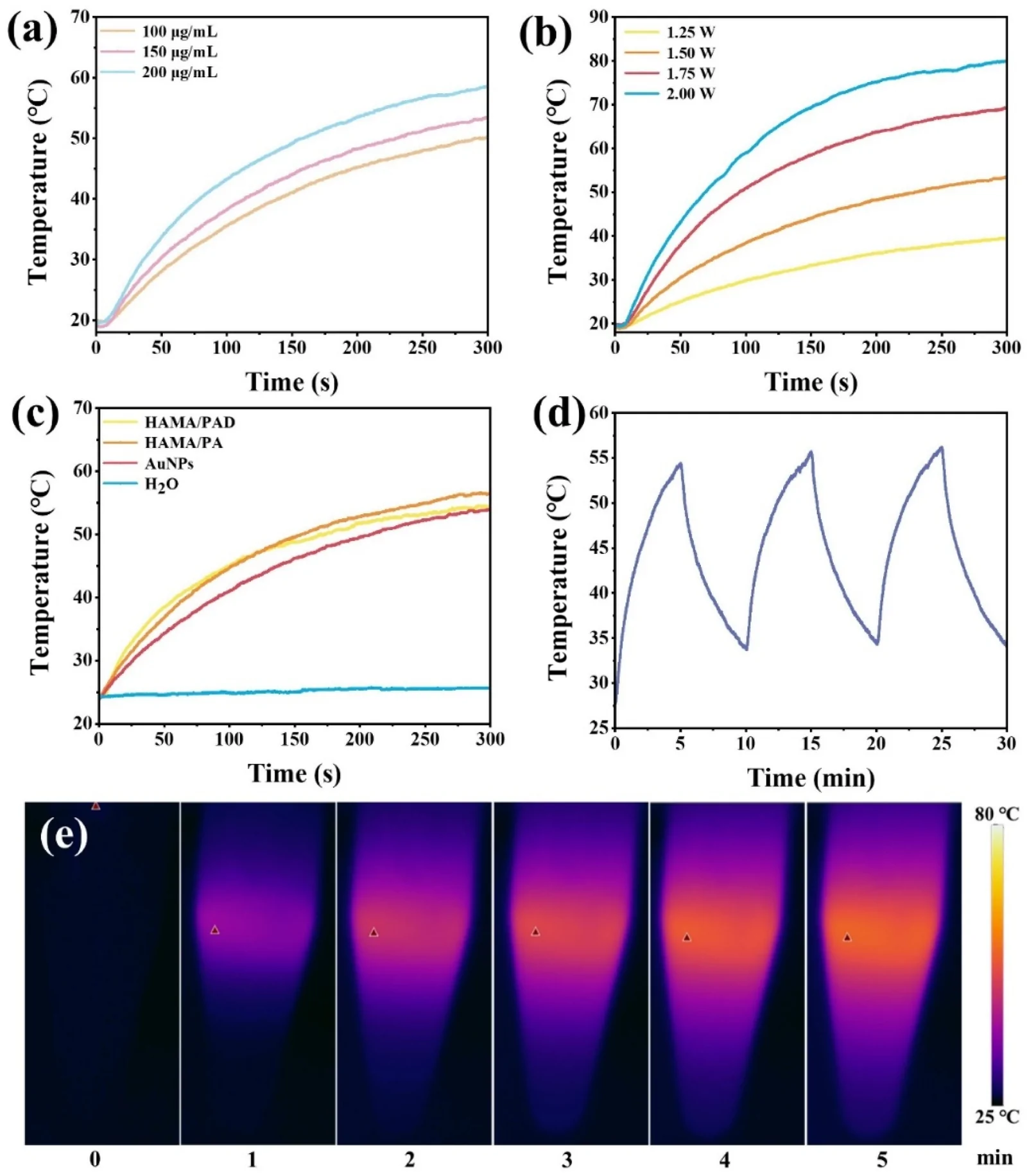
(a) Temperature profiles of AuNP dispersions at different concentrations during 808 nm NIR irradiation (1.5 W cm^−2^). (b) Photothermal heating curves of AuNP dispersions (150 µg mL^−1^) irradiated with various laser power densities. (c) Changes in sample temperature under continuous 808 nm laser irradiation (1.5 W cm^−2^). (d) Photothermal stability of the HAMA/PAD hydrogel. (e) Infrared thermal images of the HAMA/PAD hydrogel during laser irradiation.

These results confirm that both AuNP concentration and NIR irradiation intensity can be used to regulate the magnitude of photothermal heating. Such controllability is important for therapeutic applications because excessive hyperthermia may cause nonspecific damage to surrounding tissues, whereas insufficient heating may reduce tumor-cell killing. Therefore, establishing an appropriate irradiation condition is essential for achieving effective but controlled tumor PTT. Importantly, the concentration and power-dependent behaviour of AuNPs provides a tunable photothermal component for the subsequent HAMA/PAD hydrogel system, in which localized NIR heating can be combined with stimulus-responsive DOX release to enhance the overall therapeutic effect.

Continuous exposure to 808 nm NIR light at 1.5 W cm^−2^ was employed to characterize the photothermal performance of each formulation, and the result is shown in Fig. 5c. The temperature of water remained close to 25 °C throughout the irradiation period, whereas the AuNP, HAMA/PA, and HAMA/PAD groups reached approximately 54, 55, and 56 °C, respectively, within 5 min. The comparable heating profiles of free AuNPs, HAMA/PA, and HAMA/PAD indicate that sequential assembly with PNSs, DOX-SH conjugation, and incorporation into the HAMA matrix did not markedly compromise the photothermal heating capability of the AuNPs. Infrared thermal images of HAMA/PAD further showed a progressive increase in temperature with irradiation time (Fig. 5e), consistent with the temperature–time profiles and confirming the NIR-responsive heating behaviour of the composite hydrogel.

Photothermal stability was assessed by monitoring the temperature response during three repeated NIR irradiation and cooling cycles (Fig. 5d). The maximum temperature remained within approximately 54–56 °C during all three cycles, with no appreciable attenuation in the heating response. These results demonstrate that the AuNP-containing HAMA/PAD hydrogel maintained stable and reproducible photothermal performance under repeated NIR irradiation, supporting its potential application as a localized photothermal component in the combined chemo-photothermal treatment platform.

### 3.6 *In vitro* biocompatibility evaluation

Cytocompatibility is an essential consideration for the development of injectable hydrogel-based therapeutic systems, particularly when nanoscale photothermal and chemotherapeutic components are incorporated.

As shown in Fig. 6a, calcein-AM/PI double staining revealed predominantly green fluorescence in L929 fibroblasts across the control, NIR, HAMA/PNS, HAMA/PA, HAMA/PAD, and HAMA/PAD + NIR groups, with only a small number of PI-positive cells. No obvious changes in cell morphology or substantial increase in cell death were observed, indicating favorable cellular compatibility under the tested conditions. Consistent with the fluorescence observations, MTT assays showed that L929 cell viability remained above 90% following exposure to AuNPs at concentrations ranging from 0 to 250 µg mL^−1^ for 24, 48, and 72 h (Fig. 6b). Even after 72 h of exposure to 250 µg mL^−1^ AuNPs, cell viability remained approximately 92–93%, suggesting that the AuNP concentration range used for photothermal evaluation did not produce substantial cytotoxicity toward fibroblasts.

**Fig. 6:**
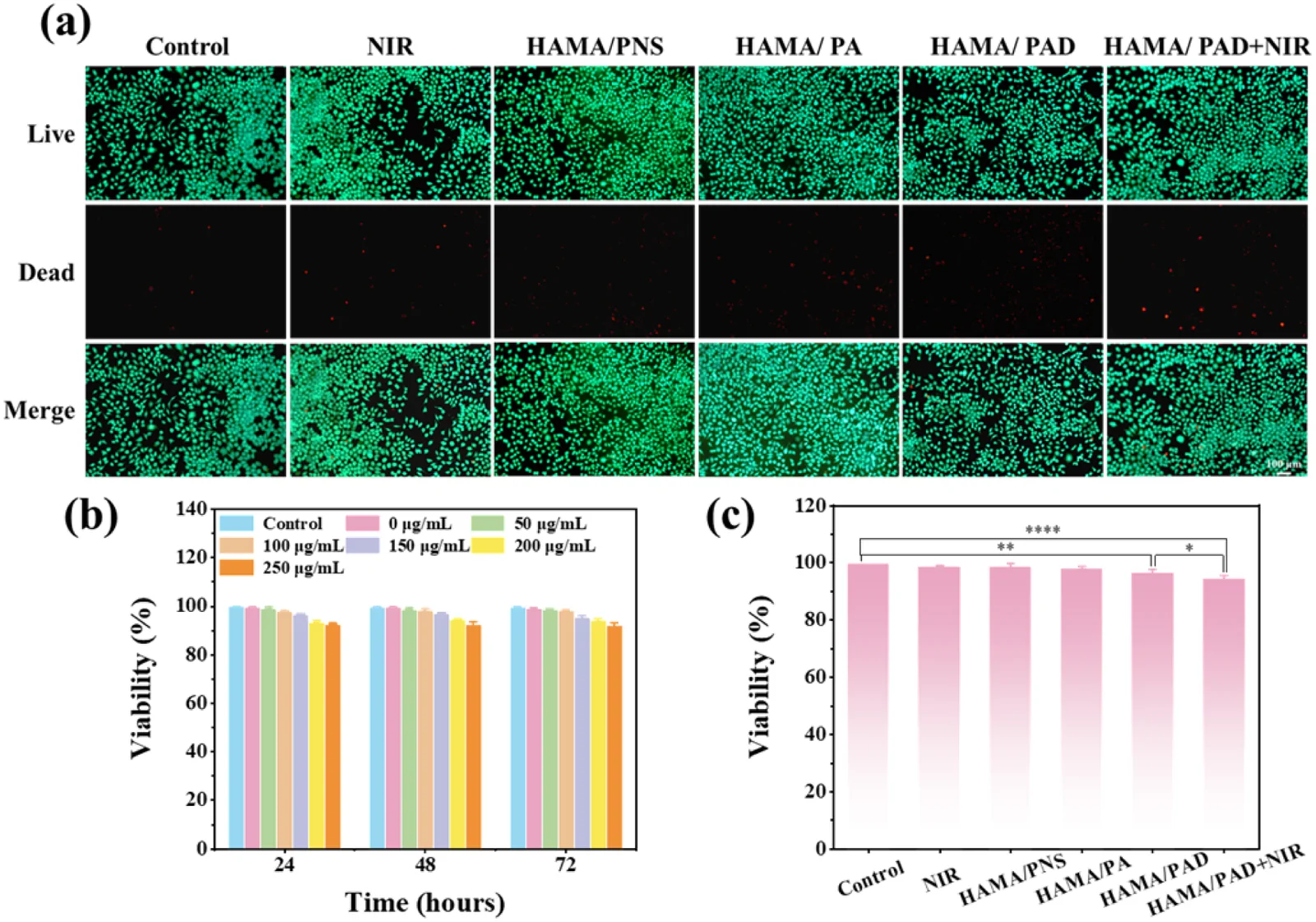
(a) Representative fluorescence micrographs of L929 cells stained with calcein-AM (green) and PI (red) to distinguish viable and nonviable cells, respectively (scale bar = 100 µm). (b) MTT-based viability of L929 cells exposed to varying concentrations of AuNPs for 24, 48, and 72 h. (c) Viability of L929 cells following incubation with the indicated formulations for 72 h, determined using the MTT assay. Data are presented as mean ± SD (*n* = 3). Statistical analysis was performed using one-way ANOVA followed by Tukey's *post hoc* test (*p* < 0.05).

The cytocompatibility of the hydrogel formulations was further assessed by MTT assay (Fig. 6c). Cell viability remained above 94% after 72 h treatment with all tested hydrogel formulations, including HAMA/PNS, HAMA/PA, HAMA/PAD, and HA-MA/PAD + NIR. Notably, the HAMA/PAD + NIR group maintained a viability of approximately 94%, indicating that the NIR irradiation conditions used for photothermal treatment did not cause pronounced indirect cytotoxicity under the experimental conditions. The comparable viability among the different formulations further suggests that incorporation of PNSs, AuNPs, and PAD into the HAMA network did not substantially compromise the biocompatibility of the hydrogel matrix.

Based on these results, we suggest that the HAMA/PAD system showed favorable *in vitro* cytocompatibility toward L929 fibroblasts. Importantly, the combination of high fibroblast viability and effective NIR-induced photothermal heating provides a useful safety-function balance for further investigation of HAMA/PAD as a localized chemo-photothermal therapeutic platform.

### 3.7 *In vitro* antitumor activity of HAMA/PAD

Murine 4T1 breast cancer cells were employed to examine the *in vitro* antitumor performance of the HAMA/PAD platform. Calcein-AM/PI staining (Fig. 7a) showed that the control, NIR, and HAMA/PNS groups were predominantly green, whereas the HAMA/PA group exhibited a modest increase in red fluorescence. In contrast, HAMA/PAD and HAMA/PAD + NIR showed markedly increased red fluorescence, with the strongest dead-cell signal observed in the HAMA/PAD + NIR group.

**Fig. 7:**
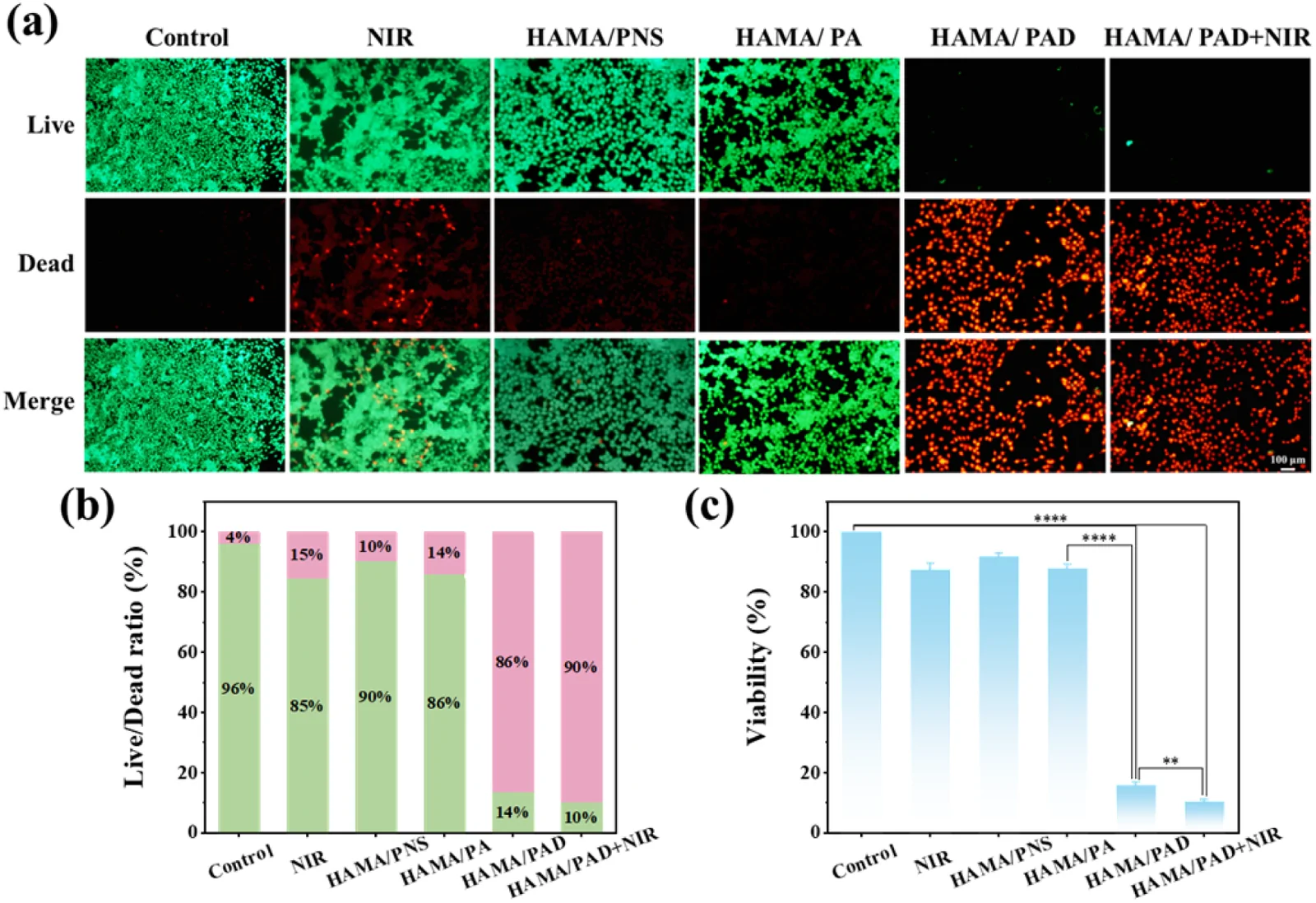
(a) Representative fluorescence images of 4T1 cells subjected to dual staining with calcein-AM (green) and PI (red), indicating viable and membrane-compromised cells, respectively (scale bar = 100 µm). (b) Quantification of viable and dead cell populations derived from the fluorescence images in (a). (c) MTT assay results showing the viability of 4T1 cells after 72 h exposure to the different formulations. Data are presented as mean ± SD (*n* = 3). Statistical analysis was performed using one-way ANOVA followed by Tukey's *post hoc* test (*p* < 0.05).

Quantitative analysis of the live/dead images showed viable-cell proportions of approximately 96%, 85%, 90%, 86%, 14%, and 10% for the control, NIR, HAMA/PNS, HAMA/PA, HAMA/PAD, and HAMA/PAD + NIR groups, respectively (Fig. 7b). Consistent with these results, MTT assays yielded corresponding cell viabilities of approximately 100%, 87%, 92%, 87%, 16%, and 10% (Fig. 7c). The pronounced cytotoxicity observed for HAMA/PAD is mainly attributable to the incorporated DOX payload, while intracellular reducing conditions may further facilitate the release of Au-bound DOX-SH after cellular internalization. HAMA/PAD combined with NIR irradiation produced the lowest cell viability (≈10%), indicating an enhanced combined chemo-photothermal effect.

Previous studies have suggested that photothermal heating may enhance tumor-cell membrane permeability and facilitate chemotherapeutic drug uptake and may also modulate MDR-associated mechanisms such as *P*-glycoprotein activity.^37,38^ These effects may contribute to the enhanced cytotoxicity observed for HAMA/PAD + NIR. The approximately 10% cell viability obtained with HAMA/PAD + NIR was comparable to that reported for our previous PNF-based O-HAMA/PGDC + NIR system under its respective experimental conditions.^21^ These findings further support the feasibility of 2D PNSs as scaffolds for constructing multifunctional chemo-photothermal platforms.^27,39^

## 4 Conclusions

In summary, an injectable HAMA/PAD composite hydrogel was developed by integrating Fmoc-FKKGSHC-derived 2D PNSs, AuNPs, and DOX-SH within a photo-crosslinked HAMA matrix. The 2D PNSs served as multifunctional molecular scaffolds for AuNP immobilization and subsequent Au–S-mediated conjugation of DOX-SH, enabling the hierarchical organization of peptide, inorganic, and therapeutic components within a single nanoscale conjugate. The resulting HAMA/PAD hydrogel exhibited an interconnected porous architecture, favorable mechanical properties and shear-thinning behaviour, together with efficient incorporation of the DOX-containing component, with an EE of 92.69 ± 1.61% and an LC of 26.28 ± 0.34%. Importantly, the hydrogel demonstrated pH- and GSH-dependent DOX release, while the incorporated AuNPs provided stable NIR-responsive photo-thermal heating at approximately 55 °C over repeated irradiation cycles. *In vitro*, HAMA/PAD showed favorable cytocompatibility toward L929 fibroblasts, whereas the combination of HAMA/PAD and NIR irradiation reduced 4T1 breast cancer cell viability to approximately 10%, demonstrating a pronounced chemo-photothermal effect.

Overall, this work demonstrates a PNS-enabled strategy for constructing an injectable nano-to-macro therapeutic architecture that integrates localized drug delivery, stimulus-responsive CT, and externally triggered PTT. The combination of molecularly organized Au–S drug conjugation and an injectable HAMA depot provides a distinctive approach for spatially confined and potentially controllable cancer treatment. An additional consideration for future *in vivo* application is the long-term biological fate of the degraded peptide assemblies and the inorganic AuNP component. The biological fate of these components may differ substantially. Progressive degradation of the peptide/HAMA matrix is expected to alter the size and physicochemical state of the released peptide assemblies, which may in turn influence their tissue distribution and elimination. In contrast, the approximately 20–30 nm AuNPs used in the present system may exhibit slower biological clearance if released as intact nanoparticles and may therefore be susceptible to longer-term tissue retention. Their eventual biodistribution and excretion are also likely to depend on whether Au is released as intact nanoparticles, smaller Au-containing species, or protein-associated complexes. Therefore, future *in vivo* studies should systematically evaluate the size evolution of the released peptide assemblies, Au biodistribution, tissue accumulation, pharmacokinetics, excretion, and long-term local and systemic safety.

## Conflicts of interest

There are no conflicts to declare.

## Supplementary Material

RA-016-D6RA08612H-s001

## Data Availability

The data that supports the finding of this study are available within the article. Supplementary information (SI) is available. See DOI: https://doi.org/10.1039/d6ra08612h.
