# Supplementary material for: Peptide–AuNP–drug conjugate-integrated photocrosslinkable hydrogel with pH/glutathione dual-responsive drug delivery and NIR-triggered heating for combined chemo-photothermal treatment
Source: RSC Adv. 2026 Oct 12;16(47):62318–29. doi: 10.1039/d6ra08612h (PMC13656027; doi:10.1039/d6ra08612h)
Supplement: Supplementary file 1 [file RA-016-D6RA08612H-s001.pdf]

Supplementary Information (SI) for RSC Advances.
This journal is © The Royal Society of Chemistry

Peptide–AuNP–drug conjugate-integrated photocrosslinkable hydrogel with pH/glutathione dual-responsive drug delivery and NIR-triggered heating for combined chemo-photothermal treatment

Ranran Wang,[a,b,c] Xinyue Wang,[b,c] Junyu Yi,[c,d] Yunyan Shi,[a] Gang Wei,[c*] and Qie Guo [a *]

[a] Department of Pharmacy, The Affiliated Hospital of Qingdao University, 266003 Qingdao, China

[b] College of Chemistry and Chemical Engineering, Qingdao University, 266071 Qingdao, China.

[c] School of Polymer Science and Engineering, Qingdao University of Science and Technology, Qingdao 266042, China

[d] Institute of Biomedical Engineering, College of Life Science, Qingdao University, 266071 Qingdao, China

Correspondence: wei-lab@qust.edu.cn or gweivincent@hotmail.com (G.W.), Tel.: +86-15066242101; guoqie822a@qdu.edu.cn (Q.G.)

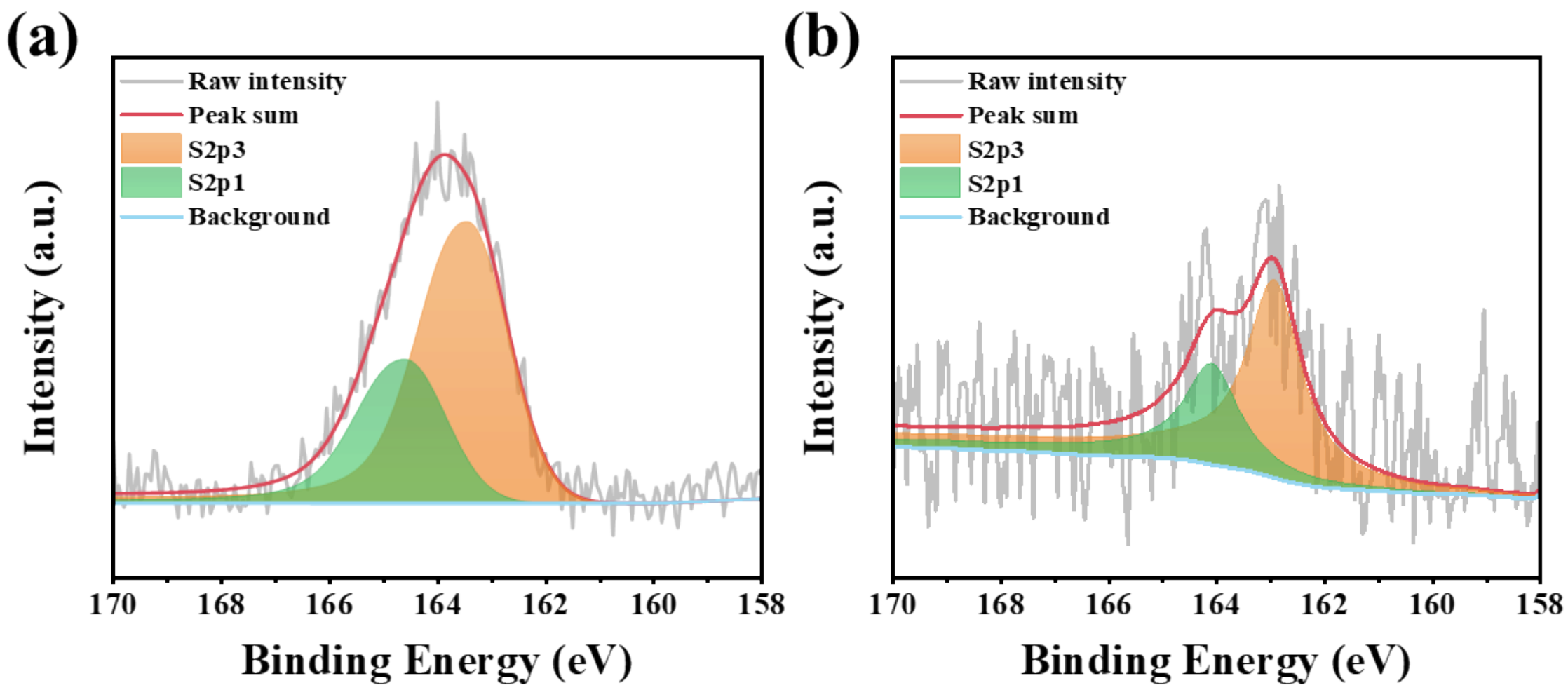

Fig. S1 (a) High-resolution S 2p spectrum of DOX-SH. (b) High-resolution S 2p spectrum of

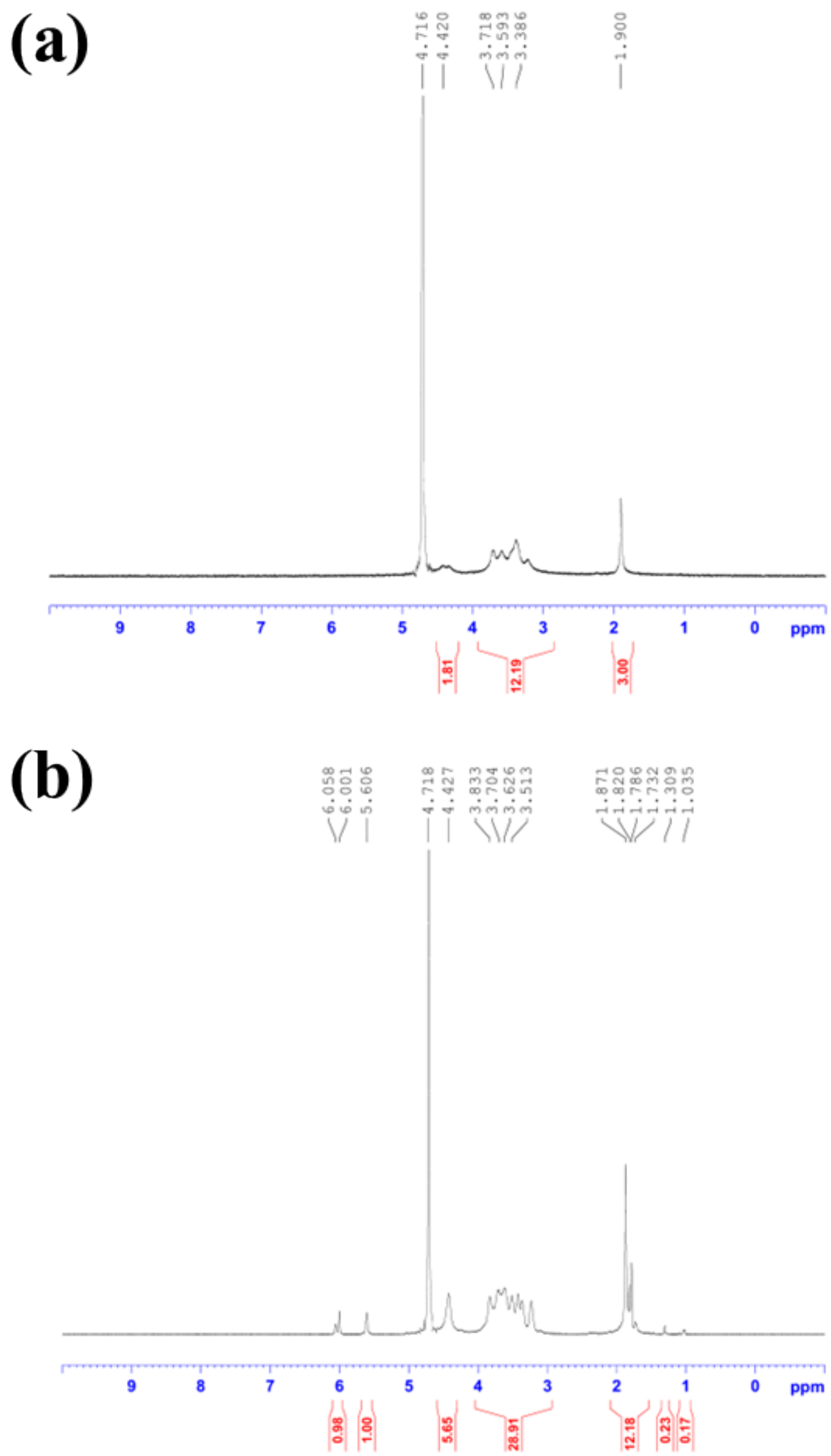

Fig. S2 ^{1}H NMR characterization of HA and HAMA in D_2O. (a)HA. (b) HAMA.

Table S1. Drug-loading quantification of three independently prepared PAD batches based on UV–vis analysis of the unbound DOX-containing fraction in the centrifugation supernatant.

Batch	A_{480}	EE (%)	LC (%)
1	0.0591	94.51	26.66
2	0.0671	91.46	26.02
3	0.0654	92.1	26.16
Mean ± SD	—	92.69±1.61	26.28±0.34
